# Effects of Non-Symbolic Approximate Number Practice on Symbolic Numerical Abilities in Pakistani Children

**DOI:** 10.1371/journal.pone.0164436

**Published:** 2016-10-20

**Authors:** Saeeda Khanum, Rubina Hanif, Elizabeth S. Spelke, Ilaria Berteletti, Daniel C. Hyde

**Affiliations:** 1 Center for Counseling and Career Advisory (C3A), National University of Science and Technology (NUST), Islamabad, Pakistan; 2 National Institute of Psychology, Quaid-i-Azam University, Islamabad, Pakistan; 3 Department of Psychology, Harvard University, Cambridge, Massachusetts, United States of America; 4 Department of Psychology, University of Illinois at Urbana-Champaign, Champaign, Illinois, United States of America; Universita degli Studi di Padova, ITALY

## Abstract

Current theories of numerical cognition posit that uniquely human symbolic number abilities connect to an early developing cognitive system for representing approximate numerical magnitudes, the approximate number system (ANS). In support of this proposal, recent laboratory-based training experiments with U.S. children show enhanced performance on symbolic addition after brief practice comparing or adding arrays of dots without counting: tasks that engage the ANS. Here we explore the nature and generality of this effect through two brief training experiments. In Experiment 1, elementary school children in Pakistan practiced either a non-symbolic numerical addition task or a line-length addition task with no numerical content, and then were tested on symbolic addition. After training, children in the numerical training group completed the symbolic addition test faster than children in the line length training group, suggesting a causal role of brief, non-symbolic numerical training on exact, symbolic addition. These findings replicate and extend the core findings of a recent U.S. laboratory-based study to non-Western children tested in a school setting, attesting to the robustness and generalizability of the observed training effects. Experiment 2 tested whether ANS training would also enhance the consistency of performance on a symbolic number line task. Over several analyses of the data there was some evidence that approximate number training enhanced symbolic number line placements relative to control conditions. Together, the findings suggest that engagement of the ANS through brief training procedures enhances children's immediate attention to number and engagement with symbolic number tasks.

## Introduction

### Overview

From birth, humans can represent and compare approximate numerical magnitudes of sets of objects [1–3]. This ability appears to be evolutionarily ancient and widely advantageous to survival and fitness, as it is shared with a wide variety of non-human animals ranging from fish to non-human primates [4–10]. Because it is manifest and shows the same signature limits in a variety of tasks (in particular, a ratio limit on the precision of the numerical contrasts that it serves to detect), the ability is thought to depend on a unitary and ancient cognitive system: the Approximate Number System (hereafter, the ANS). In contrast to other animals, humans also acquire a culture-specific symbolic number system that allows them to move beyond approximate number [11–13]. Uniquely human symbolic numerical abilities are hypothesized to arise in part from more primitive intuitions of approximate numerical magnitudes [9,14].

Recently, a small number of training experiments increased the plausibility of this hypothesis by providing initial evidence of causal effects of approximate numerical training with arrays of dots on symbolic numerical abilities, both in adults and in elementary school children [15–17], but the reliability, generality, and nature of these training effects are not known. Here we investigate the generality and robustness of the role of ANS representations on symbolic addition of written numbers, by extending the laboratory-based experimental training paradigm used in the U.S. by Hyde, Khanum, & Spelke [15] to a non-Western classroom setting in Pakistan. We also probe the nature of the effects of ANS training by testing for ANS training effects on a different symbolic numerical task that is fundamental to measurement: the mapping of symbolic numbers to points on a line.

### Background

Initial evidence for a link between exact, symbolic mathematics and approximate, non-symbolic numerical abilities was established through studies of adults, showing signatures of the ANS on symbolic number tasks (for a review see [18]). For example, when asked to compare two symbolic numbers, adults are slower and more error prone if those numbers are close in value (e.g. 65 vs. 71) than if they are farther apart (e.g. 65 vs. 91), [19–24]. Symbolic distance effects mirror non-symbolic number comparison performance and suggest an influence of approximate number on exact, symbolic number comparison [23, 25–30]. Additional evidence for an association between symbolic and non-symbolic number systems comes from neuroimaging studies showing common activation in the intraparietal sulcus for symbolic and non-symbolic numbers [18, 25, 31–35].

Other recent studies using correlational methods have revealed that individual differences in the precision of the ANS correlate with individual differences in symbolic mathematical abilities [36–41]. Some of these studies show cross-sectional correlations between ANS acuity and mathematics achievement scores at a single time point, whereas others show that precision of the ANS postdicts or predicts mathematics achievement scores [36–38]. For example, one study showed that numerical discriminations between dot arrays at 14 years of age correlated with previously obtained mathematics achievement scores throughout elementary and intermediate schooling [36]. Other studies show that children with developmental dyscalculia (i.e., extreme difficulty learning symbolic mathematics) exhibit very poor ANS acuity [39–40]. Most dramatically, individual differences in infants' sensitivity to changes in approximate number have been shown to predict preschool symbolic number abilities [41]. Given the correlational nature of these data, however, causation cannot be established. Furthermore, some investigators have failed to find a relationship between the ANS and symbolic mathematics abilities (e.g., [42–45]) and others have argued that the correlations that have been found are driven by non-numerical factors, including the capacity for inhibitory control (e.g., [46–47]).

To further probe the causal role of the ANS in mathematics performance, researchers have begun to employ experimental training paradigms (for a review see [48]). Several experiments on adults have shown that relatively short-term intensive practice with approximate number tasks result in changes in both approximate, non-symbolic number and symbolic mathematics performance [16–17, 49–50]. Training studies of adults do not reveal, however, whether the ANS influences the performance of children who are still learning mathematics.

More recently, researchers have used experimental training paradigms in child populations where conceptual understanding and fluidity of basic mathematics is still developing (for a review see [48]). One of these studies, by Hyde et al. [15] serves as the basis for the present experiments. Six- to 7-year-old children who briefly engaged the ANS by performing either an approximate numerical addition task or an approximate numerical comparison task performed better on a subsequent written test of exact, symbolic addition, relative to children who practiced either an approximate spatial addition task (adding line lengths) or a brightness comparison task. No such gain was found on a written test of reading or on a test of non-symbolic numerical acuity, suggesting a specific, and potentially causal, role for numerical magnitude representations in symbolic arithmetic [15]. Another study similarly showed that brief practice (~ 8 minutes) with approximate number comparisons that start off easy and get gradually harder lead to short-term improvements in ANS precision and superior performance in symbolic math (TEMA-3), relative to children trained with problems that initially are difficult and then become progressively more easy [50].

These findings raise a number of questions regarding the nature of these training effects and the conditions that drive them. One question is whether the effects have to do with a relationship between the ANS and mathematics at all. Cognitive training may induce in participants specific expectations concerning its effects without actually being effective [51]. For example, adults who practice training tasks that have been marketed as preventing age-related cognitive decline may show better performance on subsequent cognitive tasks not because such training is genuinely effective but because they believe that it is effective: a belief that increases their confidence or motivation. Such placebo effects could occur in studies of ANS training, if adults and children believe that the training they are given will enhance their performance on the symbolic numerical test. While, to our knowledge, tests for expectations regarding training have not been conducted with adults, one recent study of ANS training expectations was conducted with children [52]. In this study, children were familiarized with the outcome tests of symbolic arithmetic and ANS acuity used in the Hyde et al.’s training study with children [15]. Then they were introduced to the four experimental training and control tasks of that study and asked how well they thought they would perform on the two outcome tests with each type of training. Children expected all four training conditions to have equal effects, and that all would enhance children’s ANS acuity whereas none would enhance their symbolic arithmetic performance: patterns that differed markedly from what was found in the actual training study. Thus, the findings of this ANS training experiment, at one age (6–7 years), are not likely due to placebo effects.

Even if one accepts that ANS training truly influences mathematics, the question of why still remains. Because symbolic arithmetic engages the ANS, it is possible that increased precision in the ANS causes enhanced performance on symbolic arithmetic (see [16–17]). In support of this view, Park and Brannon [16] showed that the extent of change in ANS acuity over training was associated with the magnitude of the training benefit for symbolic arithmetic in adults. This view, however, is not supported by data from children showing enhanced symbolic addition after ANS training without any change in ANS acuity [15].

A second possibility is that training or practice with the ANS changes the precision of the mapping between the ANS and symbolic numbers and that the increased effectiveness of this mapping enhances symbolic arithmetic. Some correlational findings are consistent with this possibility (e.g., [53–55]), but it has not been investigated experimentally, as no measures of the symbolic number-ANS mapping have been administered after ANS training.

A third possibility is that ANS training increases participants' attention to and engagement with number in symbolic mathematical tasks, either directly (because the training task requires a focus on number) or indirectly (because the training task exercises general cognitive capacities, such as inhibitory control or working memory, that enhance symbolic arithmetic performance). These possibilities are neither exhaustive nor mutually exclusive, as ANS training may have multiple effects on performance of symbolic mathematical tasks. Each possibility therefore warrants investigation.

The reliability and external validity of ANS training effects on symbolic number abilities also are not clear. Only a few instances of ANS training effects have been reported and all of these have been conducted with upper-middle class participants in a laboratory setting in the U.S., where interest in the topic, motivation to succeed, socio-economic status, access to technology, and parental education of children are likely very high. Thus, it is yet to be determined if the core training effects can be replicated outside the laboratory, or if they are found in children who are less advantaged or live in other cultures.

### Current study

To further understand reported causal effects of the ANS on symbolic numerical abilities in children, we conducted experimental training studies in a non-Western population of elementary school children in Pakistan. Pakistani children provide an interesting test case for the generalizability of ANS training, as their country has low literacy rates and a low level of public investment in education, relative to other countries [56–58]. Furthermore, school children in Pakistan have less access to educational technology such as computers and educational software compared children in the U.S. [59]. The cultural and economic situation of Pakistani children therefore differs from that of the children typically tested in ANS training studies. In the first experiment, we used the methods of Hyde and colleagues [15] to test the effects of brief ANS practice on subsequent symbolic addition and ANS acuity in a primary school in Islamabad, Pakistan. A replication of Hyde et al.’s [15] ANS training effects in this sample and school context will begin to determine whether the ANS training effects on symbolic arithmetic are robust and general over variations in the cultural, socioeconomic, and motivational environments of children, as well as variations in the context in which they are tested (laboratory vs. school). In a second experiment, we tested the potential transfer of the effects of ANS training to a test of ANS-symbolic number mapping: a symbolic number line placement task. If the mechanism that drives enhanced symbolic number performance in ANS training is an increase in the precision of the ANS/symbolic number system mapping, then ANS training may enhance performance in symbolic number line placement, compared to control training conditions.

## Experiment 1

### Materials and Methods

#### Participants

Sixty-three total children in the 1^st^ grade of public schools in Islamabad, Pakistan were included in the final analysis. Children were quasi-randomly assigned to two experimental training groups, with the constraint that groups were initially matched for age and gender. Twenty-one of the children who participated were excluded from the final analysis for not completing all training and test measures in the study (n = 17), for failure to understand the instructions (n = 2), or because of equipment malfunction resulting in a loss of data (n = 2). The final dataset was comprised of 42 children (*M* age = 6 years, 5 months, 28 days, *SD* age = 87 days; Age Range = 6 years, 0 months, 6 days to 6 years, 11 months, 8 days; The birthdate for one participant was missing and, as such, was not included in the analysis of age). It included 19 females and 23 males. Training groups did not differ in age (*t*(39) = -0.58, *p* = .565, numerical training group: M age = 6 years, 5 months, 19 days; line length training group M age = 6 years, 6 months, 5 days) or sex (line length group: 13 males/10 females; numerical training group: 10 males/9 females; *X*^2^(1, N = 42) = 0.06, *p* = .801).

The study was approved by the National Institute of Psychology at Quaid-i-Azam University and the Federal Directorate of Education in Islamabad, Pakistan. Consent to perform the study at schools was given by the principal and head mistress of primary section school and classroom teachers. Parents and child participants gave informed written consent.

#### Design and procedure

Testing was conducted in an elementary school in Islamabad, Pakistan, with each child tested individually in a quiet room near the classroom. The experiment was introduced as an interleaved game, where children would complete tasks on a laptop computer using a special 2-response keyboard and symbolic arithmetic tests with paper and pencil (see S1 Appendix). More specifically, children were given 50 trials of the assigned computer-based training task, 20 paper and pencil symbolic addition problems, an additional 10 trials of computer-based training, and 20 more written symbolic addition problems. Finally, at the end of the procedure, all children completed a test of non-symbolic approximate numerical acuity using the computerized Panamath game. This interleaved procedure was used both to avoid boredom during testing and maximize comparability to the lab-based experiment it seeks to replicate [15]. The procedure, design, and materials were identical to those of two of the four conditions presented in the study conducted in the United States by [15], except that the experiment was presented in Urdu, the native language of the participants.

**Fig 1:**
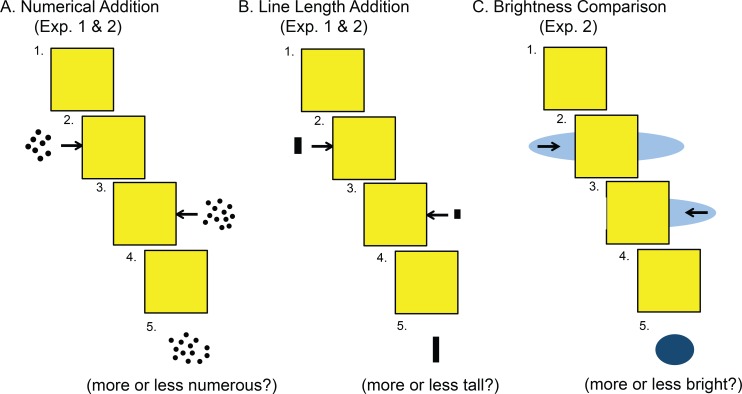
Schematic depiction of magnitude training conditions in Experiments 1 & 2. Numbers depict important time points beginning from the presentation of the first array (1) to the end of the trial (5).

***Training tasks*:** Children were assigned to one of two the non-symbolic, approximate addition training tasks used by Hyde et al. [15], one focused on numerical addition of arrays of dots and the other focused on spatial addition of line lengths (see Fig 1). In the numerical addition task, participants first saw a yellow occluder in the middle of the computer screen. Next, a dot array (addend 1) appeared on the left side of the screen and then quickly moved behind an occluder. A second dot array (addend 2) then appeared on right side of the screen and quickly moved behind the occluder. After a short pause, the occluder disappeared to reveal a third dot array (foil). Participants were asked to estimate the sum of the first two arrays and compare it with the third so as to determine whether the third array contained more or fewer dots than the sum of the previous two arrays. The line length addition training task involved the same sequence of events, but the arrays of dots were replaced by vertical lines. Children were asked to estimate the total length of a line composed of the two vertical line lengths (addends 1 and 2) if they were brought together and placed end to end, and to compare the estimated sum to the length of the third line (foil), indicating whether the third line was longer or shorter than the sum of the previous two lines (see Fig 1, also see S1 Appendix). Instructions included 8 practice problems of the assigned training task completed together with the experimenter giving feedback. The actual experiment was composed of 60 training trials, 30 trials in which the foil and the actual sum differed by a ratio of 7:4 and 30 trials in which they differed by a ratio of 7:5. Order of presentation of the comparison ratios was blocked. Training started with 25 trials at a 7:4 ratio, then 25 trials at a 7:5 ratio, followed by 5 more trials at 7:4 ratio and then 5 more trials at 7:5 ratio. Furthermore, the first 50 trials were separated from the last 10 trials by symbolic addition test problems (see below for details). Feedback in the form of tones (correct/incorrect) was explained during practice trials and given on all training trials. Stimulus presentation and recording of behavioral data (reaction time and accuracy) of the computer-based training tasks were controlled through E-prime Software (PST, Pittsburgh, PA, USA). Training trials on which children took more than 5 seconds to respond were scored as incorrect and assigned a maximum reaction time of 5000 ms. Mean reaction time and accuracy (proportion correct) were calculated for each ratio separately for each participant.

***Symbolic addition tests*:** Over the entire experiment, children were given 4 test sets of 10 symbolic addition problems on paper for a total of 40 problems. Problems were 1–3 digit addition problems presented in vertical form. Each set became progressively more difficult both within and between sets, creating 4 levels of difficulty (all problems used can be found in S1 Appendix). Before each set, children were given a sample problem to make sure they understood that exact answers were required (as opposed to approximate answers given in the training task). Children were instructed to complete the symbolic addition problems to the best of their ability. Speed on symbolic addition problems was recorded by the experimenter using a handheld stopwatch. No special verbal emphasis was given to speed over verbal accuracy, but the simultaneous recording of speed may have led participating children to weight speed over accuracy. Speed measures were confirmed through video recordings of the sessions; accuracy was calculated as the percentage of correctly answered problems, relative to incorrect or unanswered problems.

***Approximate number acuity test*:** Children’s non-symbolic, approximate numerical acuity was measured through an early version of the now freely available Panamath software ([36], www.panamath.org). This computer-based task required children to compare two collections of dots (4–15 dots in each collection) to determine which was more numerous. The task involved 6 practice trials followed by 60 test trials at 4 different difficulty levels (15 trials per bin- 2:1, 3:2, 4:3, 6:5). Dot collections appeared on the screen for up to 2000 ms. Each trial required children to indicate which set of dots was more numerous by pressing a key corresponding to the side with more dots. Children were given as much time as they wanted to respond and were given auditory feedback on their performance (an intuitive ping sound for correct responses and a bass sound for incorrect responses) for all test trials. A Weber fraction (w) was calculated as an estimate of ANS acuity on the basis of children's performance in accord with the algorithms given at www.panamath.org.

#### Analysis

Following [15], we compared the groups on age, training task performance, test performance, and ANS acuity. Training task performance was analyzed by separate mixed-factor ANOVAs on average reaction time and accuracy with the within-subjects factors of Ratio (2 levels, 7:4 vs. 7:5) and the between-subjects factor of Experimental Condition (2 levels: numerical addition vs. line addition). Additionally, we investigated improvement over the course of training by including Time (2 levels: first half vs. second half) as a factor. Test performance was analyzed using ANOVAs on average time to complete test sets (speed) and accuracy (proportion of problems answered correctly), with the between-subjects factor of Experimental Condition (2 levels). Greenhouse-Geisser corrected values were used in repeated measures analyses where the assumption of sphericity was violated.

***Treatment of missing data*:** Seventeen children understood the instructions but did not complete the experiment (approximate numerical training = 12; line length addition training = 5), and were excluded from analysis. The final dataset included 19 children in the approximate numerical addition training group and 23 children in the line length addition training group. There was no statistical difference in the proportion of children not completing the task between training groups (*X*^2^(1, N = 59) = 3.12 *p* = .077). This approach, to analyze groups with uneven numbers of subjects, is different from that taken in the original experiment [15], where missing data were more equally distributed between training groups and were replaced with overall means.

### Results

#### Training task results

***Accuracy*:** An ANOVA on average training accuracy with the between-subjects factor of Training Condition (Numerical Addition Training or Line Length Addition Training) and the within-subjects repeated factor of Ratio (7:4 or 7:5) revealed a main effect of Ratio (*F*(1,40) = 47.72, *p* < .001, η^2^_p_ = .54) (all other *p*s > .25). Across both training tasks, participants were more accurate on the easier ratios (7:4: *M* = .81, *SE* = .01) compared to the harder ratios (7:5: *M* = .70, *SE* = .01).

**Fig 2:**
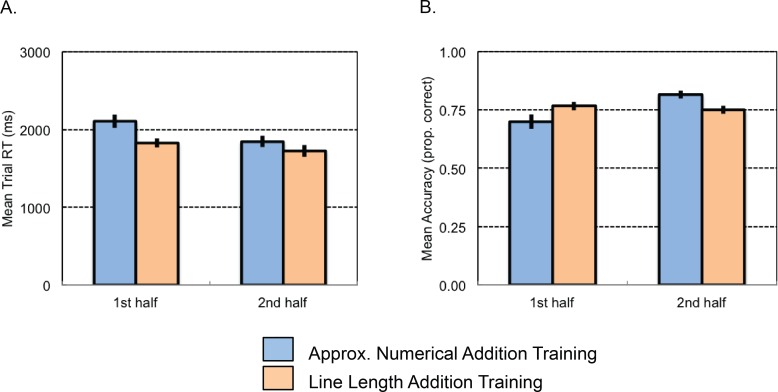
Experiment 1 training task results. Average reaction time (A) and accuracy (B) for the first (easier) and second (harder) set of training trials.

***Reaction time*:** An ANOVA on average reaction time with the between-subjects factor of Training Condition (numerical training or line length training) and the within-subjects repeated factor of Ratio (7:4 or 7:5) revealed a main effect of Ratio (*F*(1,40) = 11.72, *p* = .001, η^2^_p_ = .23) and a marginal main effect of Training Condition (*F*(1,40) = 4.24, *p* = .046, η^2^_p_ = .10). The numerical training task was performed more slowly (*M* = 1977 ms, *SE* = 74 ms) than the line-length training task (*M* = 1777 ms, *SE* = 63 ms) (see Fig 2). More generally, problems with easier ratios (most of which were performed early in training) were completed more slowly (*M* = 1961 ms, *SE* = 59 ms) than those with harder ratios (most appearing later in training) (*M* = 1774 ms, *SE* = 55 ms).

***Improvement over task*:** Despite the confounding of difficulty level with time, we attempted to analyze learning or improvement over the course of training by comparing performance on the first half of the problems at each ratio with that over the second half. To do so, we calculated accuracy and reaction time on the first half and second half of problems at each ratio level, collapsed ratios, and conducted ANOVAs with the between-subjects factor of Training Condition (numerical training or line length training) and the within-subjects repeated factor of Time (first half vs. second half).

An ANOVA on average accuracy revealed a main effect of Time (*F*(1, 40) = 6.88, *p* = .012, η^2^_p_ = .15) and an interaction between Time and Training Condition (*F*(1, 40) = 12.28, *p* = .001, η^2^_p_ = .24). Those in the non-symbolic numerical training condition showed a significant improvement between the first and second half of problems (*F*(1, 18) = 16.20, *p* = .001, η^2^_p_ = .47), whereas those in the line length training condition did not (*F*(1, 22) = 0.45, *p* = .509, η^2^_p_ = .02) (see Fig 2).

A similar analysis on reaction time revealed a main effect of Time (*F*(1, 40) = 22.23, *p* < .001, η^2^_p_ = .36), a main effect of Training Condition (*F*(1, 40) = 4.24, *p* = .046, η^2^_p_ = .10), but only a marginal interaction (*F*(1, 40) = 3.96, *p* = .053, η^2^_p_ = .09). Participants were slower on the first half of problems (*M* = 1955 ms, *SE* = 55 ms) compared to the second half (*M* = 1780 ms, *SE* = 53 ms), indicating improvement in speed or learning on the training task across both groups (see Fig 2).

#### Test results

**Fig 3:**
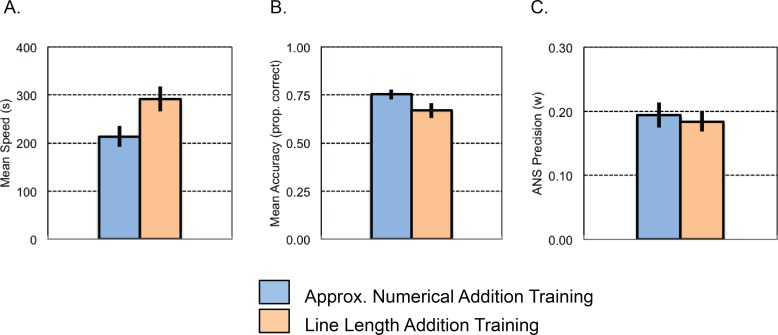
Experiment 1 test results. Average speed (A) and accuracy (B) on exact, symbolic addition problems and approximate numerical comparison performance (C) by children in the two training conditions. Note: Performance accuracy in numerical comparison (C) is represented as a Weber fraction (*w*) derived from approximate, non-verbal numerical comparison performance, where smaller *w* values correspond to more precise ANS acuity.

***Exact symbolic addition*:** An ANOVA on the speed with which participants completed the exact, symbolic addition test problems with the within-subjects factor of Difficulty (4 levels) and the between-subjects factor of Training Condition revealed main effects of Difficulty (*F*(1.11, 44.50) = 57.34, *p* < .001, η^2^_p_ = .59), and Training Condition (*F*(1, 40) = 5.01, *p* = .031, η^2^_p_ = .11), but no interaction (p = .132). More difficult symbolic addition test sets took longer (set 1 *M* = 142 s, set 2 *M* = 177 s, set 3 *M* = 242 s, & set 4 *M* = 465 s). Those participants trained on the non-symbolic numerical addition task completed the symbolic addition test sets faster (*M* = 213 s, *SE* = 22 s) than those trained on line-length addition (*M* = 292 s, *SE* = 26 s) (see Fig 3).

A similar analysis on accuracy with which participants completed the exact, symbolic addition problems revealed a main effect of Difficulty (*F*(2.46, 98.41) = 110.89, *p* < .001, η^2^_p_ = .74), with lower accuracy on more difficult problems (set 1 *M* = .92, set 2 *M* = .85, set 3 *M* = .71, set 4 *M* = .34), but no main effect or interaction with Training Condition (Training Condition: *F*(1,40) = 3.03, *p* = .089, η^2^_p_ = .070; Interaction: *F*(2.46,98.41) = 0.18, *p* = .876, η^2^_p_ = .004) (see Fig 3).

To determine whether differences in training performance between training conditions, rather than differences in content alone, influenced differences in test speed, we conducted an additional analysis of the effects of Training Condition on test speed, accounting for Training Performance (either accuracy or speed) by entering it as a covariate. These analyzes revealed that the effect of Training Condition on test speed held whether entering reaction time (*F*(1,39) = 4.11, *p* = .050, η^2^_p_ = .095) or accuracy (*F*(1,39) = 4.89, *p* = .033; η^2^_p_ = .111) on training problems as a covariate.

***Approximate number system acuity*:** No differences in the weber fraction, a measure of ANS acuity, were observed between training groups (*t*(40) = 0.42, *p* = .678)(see Fig 3).

### Discussion

Pakistani children who were randomly assigned to practice a non-symbolic approximate numerical addition task performed faster on a subsequent exact, symbolic addition test than did children assigned either to a non-symbolic line length addition task or to a brightness comparison task. The effects of speed did not result from a speed-accuracy trade-off, as the numerically-trained children were as accurate as those children trained on the other two tasks.

Because we did not have pre-training measures of symbolic arithmetic ability in these children, it is important to consider alternative reasons why children in different training conditions might have shown different performance at test. First, the training groups may have differed in numerical ability at test because of the selective attrition of children with weaker numerical skills in the numerical training condition. If this were the case then children in the numerical training group should have also outperformed those in the other two training groups in the test of non-symbolic numerical acuity, as this test is also numerical and closely resembled the task on which children in the number condition were trained. Contrary to this possibility, the three training groups showed no difference in numerical acuity at test, either in the present experiment or in the previous experiments by Hyde and colleagues [15]. Second, training effects could have been produced by higher attrition of children who were less skilled or motivated to perform symbolic arithmetic tasks. If that were the case then performance of the two groups trained on arithmetic tasks–numerical addition training and line length addition training–should both have been higher than that of children trained on the brightness comparison task. Contrary to this possibility, the line length addition training group performed equally to the brightness comparison group and worse than the numerical addition training group on symbolic addition test problems. Finally, purely random factors may have led to the creation of groups that were unequal in their arithmetic at pretest. If that were the case, findings would be unlikely to replicate from one experiment to the next. Contrary to this possibility, the findings of this study replicate those that Hyde et al. [15] obtained in two independent experiments. Based on the sum of this evidence, we conclude that the differences seen at post-test are most likely to reflect genuine effects of non-symbolic numerical training on symbolic arithmetic performance.

Thus, Experiment 1 replicates and extends the findings of Hyde et al. [15] to a non-Western country where SES, cultural values, and education differ substantially. Furthermore, these results were obtained in a school setting under the supervision of teachers, where motivation is likely lower for children compared to the lab-based studies conducted previously. As such, these new results provide evidence that ANS training effects are not simply due to an isolated cultural association of children in the U.S. of approximate number with symbolic arithmetic or to the environment of lab-based studies in the U.S. Training effects of the ANS on symbolic arithmetic replicate and generalize more broadly.

## Experiment 2

What psychological factors underlie the enhancement effect seen in Experiment 1? One plausible factor that has been proposed, but has yet to be tested through experimental training, is the precision of the association between ANS representations of number and exact number symbols (e.g., [43, 54]). Approximate numerical training may automatically co-engage symbolic and non-symbolic representations of number, producing either momentary or lasting changes in the precision of the mapping between these representations. Many studies have now found that performance in positioning symbolic numbers on a number line correlates with and predicts later math achievement (e.g., [60–63]). If the enhancing effect of ANS training on symbolic numerical abilities is due to an increase in precision or strength of the ANS-symbolic number mapping, then ANS training may also lead to an enhancement in performance on a subsequent test in which children are required to place symbolic numbers onto a line in accord with the numerical magnitudes that they represent.

Here we test for this possibility by analyzing the effects of non-symbolic, approximate numerical addition training on a symbolic number line placement test. In the number line placement task, children are presented with a horizontal line that is flanked by the symbolic numbers 0 (on the left) and 100 (on the right), together with a third number of intermediate value, positioned above the line. Children are asked to indicate where on the line the third, symbolic test number should be placed. Because the number line test also may benefit from training involving the manipulation and comparison of non-numerical magnitudes, we compared the effects of the ANS addition training used in Experiment 1 on number line placement to two other training tasks: the line-length addition training task used in Experiment 1 and a non-spatial, brightness magnitude comparison task used by Hyde et al. [15]. If effects of training on number line performance are specific to numerical magnitude training, then children in the approximate numerical training condition should perform more consistently on the number line placement task than those in the two non-numerical magnitude training conditions. On the other hand, if effects of training on number line performance are more broadly influenced by manipulating and comparing spatial magnitudes, then children in the numerical and line-length training conditions should show better performance on the number line placement task than those in the brightness training condition. Finally, if ANS training does not strengthen the mapping between the ANS and symbolic numbers, or if its benefits are equivalent to those of training on magnitude tasks with no mathematical significance, then no differences should be seen across the three training groups.

### Materials and Methods

#### Participants

Seventy-two first grade children (*M* age = 6 years, 5 months, 11 days; *SD* = 98 days; Age Range = 6 years, 0 months, 26 days to 6 years, 11 months, 29 days; 33 total females and 39 males) from public schools in Islamabad Pakistan made up the final dataset used for analysis. Participants were quasi-randomly assigned to the experimental group (non-symbolic approximate addition group, n = 24) and the control groups (line length addition training, n = 24 or brightness comparison training, n = 24) with the constraint that groups were matched for age and gender as in Experiment 1 (each group had 11 females and 13 males). An additional 23 children participated but were excluded from data analysis for not completing the study (n = 16, brightness comparison = 6; numerical addition = 7; line length addition = 3), technical computer problems (n = 6), and for failing to follow the instructions (n = 1). There were no differences in mean age (*F*(2, 69) = 0.64, *p* = .531; line addition training group: *M* = 6 years, 4 months, 23 days, *SD* = 84 days; brightness comparison group: *M* = 6 years, 5 months, 23 days, *SD* = 103 days; numerical addition training group: *M* = 6 years, 5 months, 17 days, *SD* = 106 days) or the proportion of children not completing the experiment between training groups (χ^2^(2, N = 88) = 1.38, *p* = .502).

#### Design & procedure

The research design was similar to that of Experiment 1. Children were assigned quasi-randomly to one of the three training conditions (numerical addition, line length addition or brightness comparison), after which all children completed a number line placement test. Like Experiment 1, an interleaved procedure was adopted whereby children were briefly introduced to the game through 8 practice trials, after which they were given 50 trials of their assigned training task. After the first session of training, participants were given half of the number line test problems (2 sets of 12 problems each), another 10 training problems from their assigned condition, and the other half of number line test problems (2 more 12-problem sets). As in experiment 1, this interleaved procedure was used to reduce inattention during the test trials and to maximize comparability to the previous research. At the end of the procedure, participant’s approximate numerical acuity was assessed with the computer-based Panamath game as in Experiment 1.

***Training tasks*:** The non-symbolic approximate addition training and the line length addition training were the same as those used in Experiment 1. The brightness comparison condition was similar to the line length addition, except that subjects compared the brightness of an oval-shaped object from before it moved behind a square occluder to when it was revealed from behind the occluder [15]. Specifically, an oval-shaped object appeared on both sides of the occluder and then shrunk first from the left side and then from the right side to move completely behind the occluder in the middle of the screen. The occluder then disappeared, revealing a circle that was more or less bright than the original oval. Children were asked to respond through a button press whether the test circle was more or less bright than the original oval form (see Fig 1C; also see S1 Appendix).

***Number line placement test*:** A number line placement task developed by Siegler and Booth [64–65] was used to assess the consistency of children’s symbolic number placement after training. Administration of the number line task followed the instructions given by [65]. Children saw a horizontal line (23 cm long) on paper bounded by the Arabic digit 0 on the left and 100 on the right. Each problem was presented on a separate piece of paper and contained an additional target Arabic digit on the top of the page (see S1 Appendix for an example). Children were asked to estimate the position of the target on the line by drawing a mark on the horizontal line. Children received a succession of separate lines with target digits ranging from 1 to 99 to place on the line. Specifically, children where given four stacks of paper containing 12 sheets in each stack. Each sheet contained one number line placement problem. Over the entire task, the following twenty-four numbers were presented twice (for a total of 48 trials): 3, 4, 6, 8, 12, 17, 21, 23, 25, 29, 33, 39, 43, 48, 52, 57, 61, 64, 72, 79, 81, 84, 90 and 96. Individual placement problems were presented in a novel, random order for each participant with the constraint that all twenty-four unique problems were presented before any repetition of any problem. No explicit emphasis was put on speed over accuracy by the experimenter.

Our primary analysis of number line placement concerned the relative consistency of symbolic number placement between experimental training groups. To assess consistency in mappings, we analyzed the extent to which children’s placements maintained ordinality across the task. To do this, we ordered the averaged estimates (every target was repeated twice) for each target number for every child. Estimates (estimate_i_) that increased compared to the previous estimate (estimate_i_ > estimate_i-1_), were considered correct and those that were smaller compared to previous estimate (estimate_i_ < estimate_i-1_) were considered incorrect. To summarize an individual’s scores, we calculated the proportion of correctly ordered pairs of estimates (i.e., we divided the total number of correctly ordered pairs of estimates by the total number of consecutive pairs).

A secondary analysis was conducted to compare the patterning of placements between Training Conditions. Previous work has shown that children move from a more logarithmic number line placement to a more linear placement over the first few years of schooling (e.g., [66]). To determine the overall pattern of response at the group level, we fit linear and logarithmic functions over group median estimates for each target number [66] and compared, using paired-samples t-tests, the absolute residuals from both models to determine the best fitting model (i.e., the one with the smallest residuals) for each training group. To analyze the pattern of response at the level of individual participants, we fit both the linear and logarithmic model over the average estimates for each target number for each participant. We then used the model fit (R^2^) to classify each participant according to the model that best described their pattern of estimates (Children for which average placements did not conform to either pattern were excluded from this analysis: n = 1 from the numerical training condition and n = 2 from the brightness training condition). To then further analyze the effect of training condition, we compared the distribution of linear vs. logarithmic placements across and within groups using non-parametric tests.

A tertiary analysis investigated the accuracy of placements between Training Groups. Given that a majority of placements were better fit by a linear than a logarithmic model, we computed placement accuracy as the extent of absolute proportion of error (PE) from a linear placement model (PE = [(estimate—target number)/ scale of estimates]).

Finally, we analyzed number line placement speed between training groups. Speed was measured using stopwatch and confirmed through video recordings of the test sessions. More specifically, the time to complete each of the four stacks (12 problems each) was measured and summed to obtain a total completion time for each participant.

Given the potential differences in familiarity with relative positioning of smaller and large numbers, additional post-hoc exploratory analysis also examined the factor of number size (problems involving smaller numbers (≤ 50) or larger numbers (>50) on number line placement performance).

***Panamath task*:** The same Panamath task as in Experiment 1 served to measure children’s approximate non-verbal numerical acuity.

### Results

#### Training task results

**Fig 4:**
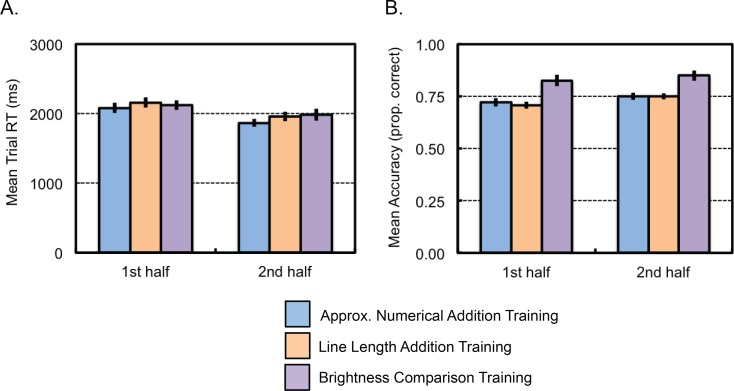
Experiment 2 training task results. Average reaction time (A) and accuracy (B) for the first (easier) and second (harder) set of training trials.

***Accuracy*:** An ANOVA on average training accuracy with the between-subjects factor of Training Condition (numerical addition training, line length addition training, or brightness comparison training) and the within-subjects repeated factor of Ratio (7:4 or 7:5) revealed a main effect of Ratio (*F*(1,69) = 38.05, *p* < .001, η^2^_p_ = .36), a main effect of Training Condition (*F*(2,69) = 11.95, *p* < .001, η^2^_p_ = .26), and an interaction between Ratio and Training Condition (*F*(2,69) = 7.63, *p* = .001, η^2^_p_ = .18) (see Fig 4). A post-hoc comparison of conditions revealed that, overall, participants were more accurate at the brightness comparison training than line length addition training (*t*(46) = 3.99, *p* < .001) and numerical addition training (*t*(46) = 3.58, *p* = .001). There was no difference between groups in accuracy for the line length training and the numerical addition training (*t*(46) = -0.28, *p* = .784). Analysis of each condition separately revealed an effect of Ratio on accuracy in the line length addition training group (*F*(1,23) = 13.35, *p* = .001, η^2^_p_ = .37) and numerical addition training group (*F*(1,23) = 45.23, *p* < .001, η^2^_p_ = .66), but not in the brightness comparison training group (*F*(1,23) = 0.53, *p* = .474, η^2^_p_ = .02).

***Reaction time*:** An analysis of reaction time revealed only a main effect of Ratio (*F*(1,69) = 5.10, *p* = .027, η^2^_p_ = .07, other *p*s > .35), with slower reaction times to more distant ratios. However, as in Experiment 1, ratio comparison was confounded with order of presentation.

***Improvement over training task*:** An similar analysis of Time (between the first half and second half of problems at each difficulty level), or practice over the course of the training, revealed that accuracy (*F*(1,69) = 6.17, *p* = .015, η^2^_p_ = .08) significantly increased and reaction time decreased (*F*(1,69) = 32.58, *p* < .001, η^2^_p_ = .32) over the second half of problems compared to the first.

#### Test results

***Ordinality of number line placements*:** Our primary analysis was concerned with the extent to which number line placements respected the ordinality of the test numbers. An analysis of ordinality revealed no significant effect of Training Condition (*F*(2,69) = 2.63, *p* = .079, η^2^_p_ = .07).

**Fig 5:**
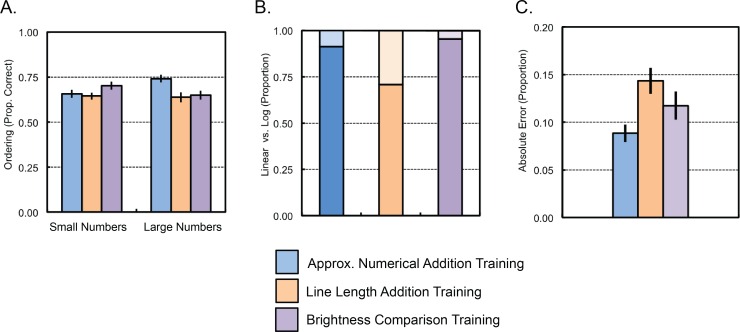
Number line placement test performance by children in the three training conditions of Experiment 2. (A) Accuracy in ordinality of children's placements. (B) Proportion of children with more linear placements (dark portion of bars) and with more logarithmic placements (lighter portion of bars). (C) Proportion of error in children’s placements, with smaller values corresponding to better performance.

A further exploratory analysis of the role of Number Size and Training Condition on ordinality of placements revealed a significant interaction between Training Condition and Number Size (*F*(2,69) = 4.84, *p* = .011, η^2^_p_ = .12, see Fig 5). Post hoc analyses of smaller and larger number placements separately revealed a significant main effect of Training Condition for larger number placements (*F*(2,69) = 5.28, *p* = .007, η^2^_p_ = .13), but not for smaller number placements (*F*(2,69) = 2.00, *p* = .143, η^2^_p_ = .06). Pairwise comparisons between Training Conditions for the larger number placements revealed that number line placements were more consistent for the non-symbolic numerical addition training group compared to the other two groups (vs. brightness comparison training group: *t*(46) = 2.78, *p* = .008; vs. line length addition training group: *t*(46) = 3.03, *p* = .004, see Fig 5). No differences in the ordinality of placements were observed between the brightness comparison training group and the line length addition training group (*t*(46) = 0.33, *p* = .740).

***Patterning of number line placements*:** Fits over group medians for each target number revealed that the data were better fit by a linear than a logarithmic pattern (line length addition training group: linear R^2^ = .978, log. R^2^ = .859, *p*s < .001, *t*(23) = -5.40, *p* < .001; numerical addition training group: linear R^2^ = .978, log. R^2^ = .820, *p*s < .001, *t*(23) = -4.78, *p* < .001; brightness comparison training group: linear R^2^ = .984, log. R^2^ = .864, *p*s < .001, *t*(23) = -5.52, *p* < .001, see Fig 5), suggesting that children’s placements on the number line were more linear than logarithmic regardless of condition.

Further analysis of the patterning of responses, classifying each child according to the better fitting model (linear or logarithmic), revealed a marginal difference in the proportion of children showing linear placements between conditions (χ^2^(2, N = 69) = 6.55, *p* = .038; but see Fisher’s Exact Test = 5.68, *p* = .065). While all groups showed more children with placements better fit by a linear model than a logarithmic model, the between-groups effect of training condition was driven by a greater proportion of placement patterns characterized as logarithmic in the line length addition group (17 linear vs. 7 logarithmic, binomial *p* = .064) compared to the numerical addition training group and the brightness comparison training group (brightness comparison training group: 21 linear vs. 1 logarithmic, binomial *p* < .001; numerical addition training group = 21 linear vs. 2 logarithmic, binomial *p* < .001, see Fig 5).

***Accuracy of number line placements*:** An analysis of accuracy, measured as the proportion absolute error in placements based on a linear model, with the between subjects factor of Training Condition (numerical addition, line addition, brightness comparison) revealed a main effect of Training Condition (*F*(2,69) = 4.65, *p* = .013, η^2^_p_ = .12, see Fig 5). Post-hoc pairwise comparisons revealed greater proportion of error in the line length addition group (*M* = .143, *SE* = .014) compared to the numerical addition group (*M* = .088, *SE* = .009; t(46) = 3.35, p = .002), with the brightness comparison group patterning in-between the other two conditions (*M* = .117, *SE* = .015, ps > .1).

A further exploratory analysis revealed no main effect of Number Size (*F*(1, 69) = 1.44, *p* = .24) or interaction of Number Size with Training Condition (*F*(2, 69) = 1.21, *p* = .31) on accuracy (brightness comparison: small numbers *M* = .115, *SE* = .018 and large numbers *M* = .121, *SE* = .016; line length addition: small numbers *M* = .154, *SE* = .017 and large numbers *M* = .129, *SE* = .013; numerical addition: small numbers *M* = .093, *SE* = .011 and large numbers *M* = .082, *SE* = .010).

***Speed of number line placements***: No significant differences between experimental training groups were observed in total time to complete number line placement problems (line: *M* = 961.3 seconds, *SE* = 127.3 seconds; number: *M* = 740.7 seconds, *SE* = 68.27 seconds; brightness comparison group: *M* = 885.3 seconds, *SE* = 124.3 seconds; line length group vs. number group: *t*(46) = 1.53, *p* = .134; number group vs. brightness group: *t*(46) = -1.02, *p* = .313; line length group vs. brightness group: *t*(46) = 0.43, *p* = .671). Since there were no differences in overall speed between groups, differential accuracy on the number line tasks between training groups observed cannot be explained by a speed-accuracy trade-off.

***Approximate number system acuity*:** No differences in the weber fraction were observed in a between training groups (*F*(2,69) = 0.85, *p* = .430; line length addition training group: *M* = .229, *SE* = .018; numerical addition training group: *M* = .189, *SE* = .028; brightness comparison training group: *M* = .201, *SE* = .020).

### Discussion

Several analyses revealed better number line placement in children trained on approximate numerical addition compared to children trained on line length addition. More specifically, number line placements were more ordinal, more accurate, and marginally more linear in children trained on approximate numerical addition compared to children trained on line length addition. However, in the case of accuracy and linearity, placements of children trained on approximate numerical addition were no different from placements of children trained on the brightness comparison task. Only in one exploratory analysis on the effects of training type on larger symbolic number (50–100) line placement problems did we find enhanced effects of ANS training compared to both the line length and the brightness magnitude training conditions. More specifically, number line placements for numbers 50–100 better maintained ordinality in children who had received approximate numerical addition training compared both to children who had received brightness training and to children who had received line length addition training. Thus, there was only partial evidence that approximate numerical training strengthened or sharpened the mapping between the ANS and symbolic numbers. Interestingly, children trained on the line length addition task performed consistently *worse* on number line placements across all metrics compared to groups of children who trained on approximate number and brightness magnitude comparison, despite the similarity of the operations on line segments required by the two tasks.

## General Discussion

Several aspects of the present study shed light on the effects of non-symbolic numerical practice on symbolic mathematics. First, Experiment 1 provides evidence that the approximate number system is causally related to symbolic arithmetic performance, as children who engaged in approximate numerical addition through a brief training exercise outperformed children who engaged in approximate spatial addition or brightness comparison on a test of exact symbolic arithmetic. This study used the same materials and procedure as a published study showing this training effect in children [15], using training tasks and outcome measures that have been shown not to engender expectations in children that could produce a placebo effect [52]. The present findings thus replicate the key training effect of that study and attest to its robustness.

Second, the current findings suggest that relationships between approximate number and symbolic mathematics in children hold outside of the laboratory. Whereas the original study of Hyde and colleagues [15] was conducted in a laboratory, the current study was conducted in an elementary school. Motivations for participation likely differ between such laboratory-based studies were children are brought into the lab one-by-one by a parent or guardian and studies conducted during the school day in a quiet corner of the classroom. Nevertheless, we observed similar effects on both groups, suggesting that the effect generalizes from laboratory to more practical (and possibly educational) settings.

Third, the effects of engagement of approximate number on symbolic arithmetic performance were observed in a group of Pakistani children, whose education and culture vary from the upper-middle class children tested in previous studies of approximate number training in the United States. For example, Pakistan as a country has one of the lowest literacy rates in the world [58] and a relatively low level of public investment in education, particular in primary school [56–58]. Such statistics suggest less cultural emphasis on and resources available for education than in the U.S., where children have been previously tested. The effects of approximate number practice on symbolic addition therefore suggest that the relationship between the approximate number system and mathematics likely generalizes to a variety of cultural and socio-economic settings. Relatedly, children in Pakistan have less access to technology like the computers used for testing than do children in the U.S. (e.g., [59]). As such, our method does not depend on having substantial experience with computers, as was more likely to be the case with U.S. children than Pakistani children. It should be noted that despite cultural differences, the current data from Pakistan are comparable in non-verbal numerical acuity (weber fractions) and levels of performance on symbolic addition to those obtained in a previous U.S sample of children.

Fourth, we found evidence for a specific enhancing effect of approximate number training on symbolic number line placement. By most metrics of precision on the number line, those in the ANS training condition performed significantly better on a subsequent symbolic number line placement task than those in the line length addition training condition. Furthermore, on the main measure of ordering consistency, children in the ANS training condition performed better than those in the brightness comparison control condition as well. Together these results suggest a causal influence of ANS training on the symbolic number-magnitude mapping. However, the fact that the ANS training group was not significantly different from the brightness comparison group on two other metrics of number line performance suggests that the effects of ANS training on symbolic number-spatial magnitude mappings may be weak. Further work is needed to investigate these effects, their sources, and their implications.

Fifth, the results of Experiment 2 raise the possibility that brief practice with continuous magnitudes (line lengths) did not improve symbolic number line placement relative to practice in the other training conditions. This finding was unexpected, given the privileged relationship that has been posited between number and spatial magnitudes (e.g., [63, 67–69]) and the similarity of the operations that are required to conjoin two segments into a single line (in the line length addition training task) and to divide a single line into segments (in the number line outcome test). This finding extends those of Hyde and colleagues [15] and of Experiment 1, and it attests to the distinctness of numerical magnitudes from other continuous magnitudes.

In sum, many studies have now shown a relationship between non-verbal numerical abilities and symbolic number and mathematics (see [69–70]). Our results accord with other recent findings to suggest that training with approximate numerical arithmetic can improve basic symbolic arithmetic in children and adults [15–17]. The fact that no differences in the acuity of the ANS were found between experimental and control training groups suggests that brief practice with approximate numerical arithmetic is not likely to change fundamentally the underlying non-symbolic representations of number. Regardless of the mechanism producing the effects of training on the tests of symbolic mathematics, it appears that simply engaging the ANS is enough to cause short-term enhancements in subsequent performance. It seems reasonable to consider approximate numerical arithmetic as an easy practice, or warm-up exercise to improve children’s engagement with the exact, symbolic arithmetic that they learn in school.

## Supporting Information

S1 Appendix Further methodological details. (PDF)

S1 Data Compressed Experiment 1 dataset files. (ZIP)

S2 Data Compressed Experiment 2 dataset files. (ZIP)

S3 Data Compressed additional data files. (ZIP)

## References

[1] Izard V, Sann C, Spelke ES, Streri A. Newborn infants perceive abstract numbers. Proc Natl Acad Sci. 2009;106(25):10382–5. 10.1073/pnas.0812142106 19520833 PMC2700913

[2] Coubart A, Izard V, Spelke ES, Marie J, Streri A. Dissociation between small and large numerosities in newborn infants. Developmental Sci. 2014;17: 11–22. 10.1111/desc.12108 PMC4624260 24267592

[3] Schleger F, Landerl K, Muenssinger J, Draganova R, Reinl M, Kiefer-Schmidt I, et al Magnetoencephalographic signatures of numerosity discrimination in fetuses and neonates. Dev Neuropsychol. 2014; 39: 316–29. 10.1080/87565641.2014.914212 24854775

[4] Brannon EM, Terrace HS. Ordering of the numerosities 1–9 by monkeys. Science. 1998; 282: 746–9. 9784133 10.1126/science.282.5389.746

[5] Brannon EM, Terrace HS. Representation of the numerosities 1–9 by Rhesus Monkeys (*Macaca mulatta*). J Exp Psychol Anim B. 2000; 26: 31–49. 10.1037//0097-7403.26.1.31 10650542

[6] Brannon EM. The independence of language and mathematical reasoning. Proc Natl Acad Sci. 2005;109: 3177–8. 10.1073/pnas.0500328102 PMC552939 15728346

[7] Cantlon J, Brannon EM. Semantic congruity affects numerical judgments similarly in monkeys and humans. Proc Natl Acad Sci. 2005; 102: 16507–11. 10.1073/pnas.0506463102 16260752 PMC1283437

[8] Nieder A, Dehaene S. Representation of number in the brain. Annu Rev Neurosci. 2009;32:185–208. 10.1146/annurev.neuro.051508.135550 19400715

[9] Feigenson L, Dehaene S, Spelke ES. Core systems of number. Trends Cogn Sci. 2004;8(7):307–14. 10.1016/j.tics.2004.05.002 15242690

[10] Piffer L, Agrillo C, Hyde DC. Small and large number discrimination in guppies. Anim Cogn. 2012;15(2):215–21. 10.1007/s10071-011-0447-9 21909934

[11] Le Corre M, Carey S. One, two, three, four, nothing more: an investigation of the conceptual sources of the verbal counting principles. Cognition. 2007;105(2):395–438. 10.1016/j.cognition.2006.10.005 17208214 PMC3880652

[12] Wynn K. Infants’ individuation and enumeration of actions. Cognition. 1990;32(2):155–93.

[13] Carey S. The origin of concepts New York: Oxford University Press; 2009.

[14] Dehaene S., Cohen L. Cultural recycling of cortical maps. Neuron. 2007;56,384–98. 10.1016/j.neuron.2007.10.004 17964253

[15] Hyde DC, Khanum S, Spelke ES. Brief non-symbolic, approximate number practice enhances subsequent exact symbolic arithmetic in children. Cognition. 2014; 131(1):92–107. 10.1016/j.cognition.2013.12.007 24462713 PMC4061922

[16] Park J, Brannon EM. Training the approximate number system improves math proficiency. Psychol Sci. 2013;24(10):2013–19. 10.1177/0956797613482944 23921769 PMC3797151

[17] Park J, Brannon EM. Improving arithmetic performance with number sense training: An investigation of underlying mechanism. Cognition. 2014;133(1):188–200. 10.1016/j.cognition.2014.06.011 25044247 PMC4145063

[18] Piazza M. Neurocognitive start-up tools for symbolic number representations. Trends Cogn Sci. 2010;14(12):542–51. 10.1016/j.tics.2010.09.008 21055996

[19] Moyer RS, Landauer TK. Time required for judgments of numerical inequality. Nature. 1967;215:1519–20. 6052760 10.1038/2151519a0

[20] Dehaene S, Dupoux E, Mehler J. Is numerical comparison digital? Analogical and symbolic effects in two-digit number comparison. J Exp Psychol Human. 1990;16: 626–41. 10.1037//0096-1523.16.3.626 2144576

[21] Dehaene S, Akhavein R. Attention, automaticity, and levels of representation in number processing. J Exp Psychol Learn Mem Cogn. 1995;21(2):314–26. 7738503 10.1037//0278-7393.21.2.314

[22] Dehaene S, Dehaene-Lambertz G, Cohen L. Abstract representations of numbers in the animal and human brain. Trends Neurosci. 1998;21(8):355–61. 9720604 10.1016/s0166-2236(98)01263-6

[23] Holloway ID, Ansari D. Mapping numerical magnitudes onto symbols: the numerical distance effect and individual differences in children’s mathematics achievement. J Exp Child Psychol. 2009;103(1):17–29. 10.1016/j.jecp.2008.04.001 18513738

[24] Van Opstal F, Gevers W, De Moor W, Verguts T. Dissecting the symbolic distance effect: Comparison and priming effects in numerical and nonnumerical orders. Psychon Bull Rev. 2008;15(2):419–25. 18488662 10.3758/pbr.15.2.419

[25] Pinel P, Dehaene S, Rivière D, Le Bihan D. Modulation of parietal activation by semantic distance in a number comparison task. Neuroimage. 2001;14(5):1013–26. 10.1006/nimg.2001.0913 11697933

[26] Lonnemann J, Linkersdörfer J, Hasselhorn M, Lindberg S. Symbolic and non-symbolic distance effects in children and their connection with arithmetic skills. J Neurolinguistics. 2011;24(5):583–91.

[27] Mussolin C, Mejias S, Noël M-P. Symbolic and nonsymbolic number comparison in children with and without dyscalculia. Cognition. 2010;115(1):10–25. 10.1016/j.cognition.2009.10.006 20149355

[28] Rousselle L, Noël M-P. Basic numerical skills in children with mathematics learning disabilities: a comparison of symbolic vs non-symbolic number magnitude processing. Cognition. 2007;102(3):361–95. 10.1016/j.cognition.2006.01.005 16488405

[29] Price GR, Holloway ID, Räsänen P, Vesterinen M, Ansari D. Impaired parietal magnitude processing in developmental dyscalculia. Curr Biol. 2007;17(24):R1042–3. 10.1016/j.cub.2007.10.013 18088583

[30] Defever E, Sasanguie D, Gebuis T, Reynvoet B. Children’s representation of symbolic and nonsymbolic magnitude examined with the priming paradigm. J Exp Child Psychol. 2011;109(2):174–86. 10.1016/j.jecp.2011.01.002 21324472

[31] Dehaene S, Piazza M, Pinel P, Cohen L. Three parietal circuits for number processing. Cogn Neuropsychol. 2003;20(3):487–506. 10.1080/02643290244000239 20957581

[32] Pinel P, Piazza M, Le Bihan D, Dehaene S. Distributed and overlapping cerebral representations of number, size, and luminance during comparative judgments. Neuron. 2004;41(6):983–93. 15046729 10.1016/s0896-6273(04)00107-2

[33] Cohen Kadosh R, Cohen Kadosh K, Kaas A, Henik A, Goebel R (2007) Notation dependent and -independent representations of numbers in the parietal lobes. Neuron; 53: 307–14. 10.1016/j.neuron.2006.12.025 17224410

[34] Piazza M, Pinel P, Le Bihan D, Dehaene S. A magnitude code common to numerosities and number symbols in human intraparietal cortex. Neuron. 2007;53(2):293–305. 10.1016/j.neuron.2006.11.022 17224409

[35] Vogel SE, Grabner RH, Schneider M, Siegler RS, Ansari D. Overlapping and distinct brain regions involved in estimating the spatial position of numerical and non-numerical magnitudes: an fMRI study. Neuropsychologia. 2013;51(5):979–89. 10.1016/j.neuropsychologia.2013.02.001 23416146

[36] Halberda J, Mazzocco MMM, Feigenson L. Individual differences in non-verbal number acuity correlate with maths achievement. Nature. 2008;455(7213):665–8. 10.1038/nature07246 18776888

[37] Mazzocco MMM, Feigenson L, Halberda J. Preschoolers’ precision of the approximate number system predicts later school mathematics performance. PLoS One. 2011;6(9):e23749 10.1371/journal.pone.0023749 21935362 PMC3173357

[38] Libertus ME, Feigenson L, Halberda J. Preschool acuity of the approximate number system correlates with school math ability. Dev Sci. 2011;14(6):1292–300. 10.1111/j.1467-7687.2011.01080.x 22010889 PMC3338171

[39] Piazza M, Facoetti A, Trussardi AN, Berteletti I, Conte S, Lucangeli D, et al Developmental trajectory of number acuity reveals a severe impairment in developmental dyscalculia. Cognition. 2010;116(1):33–41. 10.1016/j.cognition.2010.03.012 20381023

[40] Mazzocco MMM, Feigenson L, Halberda J. Impaired acuity of the approximate number system underlies mathematical learning disability (dyscalculia). Child Dev. 2011;82(4):1224–37. 10.1111/j.1467-8624.2011.01608.x 21679173 PMC4411632

[41] Star A, Libertus ME, Brannon EM. Number sense in infancy predicts mathematical abilities in childhood. Proc Natl Acad Sci. 2013; 110: 18116–20. 10.1073/pnas.1302751110 24145427 PMC3831472

[42] Castronovo J, Göbel SM. Impact of high mathematics education on the number sense. PLoS ONE. 2012;7(4): e33832 10.1371/journal.pone.0033832 22558077 PMC3338810

[43] De Smedt B, Nöel MP, Gilmore C, Ansari D. How do symbolic and non-symbolic numerical magnitude processing skills relate to individual differences in children’s mathematical skills? A review of evidence from brain and behavior. Trends Neurosci Educ. 2013;2:48–55.

[44] Holloway ID, Ansari D. Mapping numerical magnitudes onto symbols: The numerical distance effect and individual differences in children’s mathematics achievement. J Exp Child Psychol. 2009;103(1):17–29. 10.1016/j.jecp.2008.04.001 18513738

[45] Price GR, Palmer D, Battista C, & Ansari D. Nonsymbolic numerical magnitude comparison: reliability and validity of different task variants and outcome measures, and their relationship to arithmetic achievement in adults. Acta Psychol. 2012;140(1):50–7. 10.1016/j.actpsy.2012.02.008 22445770

[46] Gilmore CK, Attridge N, Clayton S, Cragg L, Johnson S, Marlow N, et al Individual Differences in Inhibitory Control, Not Non-Verbal Number Acuity, Correlate with Mathematics Achievement. PLoS One. 2013;8(6):1–9. 10.1371/journal.pone.0067374 PMC3681957 23785521

[47] Fuhs MW, McNeil NM. ANS acuity and mathematics ability in preschoolers from low-income homes: contributions of inhibitory control. Developmental Sci. 2013;16(1):136–48. 10.1111/desc.12013 23278935

[48] Hyde DC, Berteletti I, Mou Y. Approximate numerical abilities and mathematics: Insight from correlational and experimental training studies In: Cappelletti M, Fias W, editors. Progress in brain research: The mathematical brain across the lifespan. Oxford, UK: Elsevier; 2016; 227: 335–51. 10.1016/bs.pbr.2016.04.011 27339018

[49] DeWind NK, Brannon EM. Malleability of the approximate number system: effects of feedback and training. Front Hum Neurosci. 2012;6:68 10.3389/fnhum.2012.00068 22529786 PMC3329901

[50] Odic D, Hock H, Halberda J. Hysteresis affects number discrimination in young children. J Exp Psychol G. 2013; 143(1): 255–65. 10.1037/a0030825 PMC4390026 23163765

[51] Boot WR, Simons DJ, Stothart C, Stutts C. The pervasive problem with placebos in psychology: Why active control groups are not sufficient to rule out placebo effects. Perspect Psychol Sci. 2013;8(4):445–54. 10.1177/1745691613491271 26173122

[52] Dillon MR, Pires AC, Hyde DC, Spelke ES. Children’s expectations about training the approximate number system. Br J Dev Psychol. 2015; 33(4): 411–8. 10.1111/bjdp.12118 26399713

[53] Goffin C, Ansari D. Beyond magnitude: Judging ordinality of symbolic number is unrelated to magnitude comparison and independently relates to individual differences in arithmetic. Cognition. 2016; 150: 68–76. 10.1016/j.cognition.2016.01.018 26851638

[54] Lyons I, Beilock SL. Numerical ordering ability mediates the relation between number-sense and arithmetic competence. Cognition. 2011; 121: 256–61. 10.1016/j.cognition.2011.07.009 21855058

[55] vanMarle K, Chu FW, Li Y, Geary DC. Acuity of the approximate number system and preschoolers’ quantitative development. Developmental Sci. 2014; 17: 492–505. 10.1111/desc.12143 24498980

[56] Ali SW, Siddiqui F. Education in Pakistan: state of affairs at a glance Report. Islamabad, Pakistan: Manzil Pakistan, 2013 Available: http://www.manzilpakistan.org/wp-content/uploads/2013/10/Education-in-Pakistan-State-of-affairs-at-a-glance.pdf.

[57] Statistics Division, Pakistan Bureau of Statistics. Government of Pakistan. Pakistan social and living standards measurement survey (PSLM) 2014–15. Islamabad, Pakistan; March, 2016. Available: http://www.pbs.gov.pk/node/1650.

[58] United Nations Educational, Scientific and Cultural Organization (UNESCO), Islamabad. Why Pakistan needs a literacy movement. Islamabad, Pakistan; March, March, 2012. Available: http://unesco.org.pk/education/documents/publications/Why_Pakistan_Needs_Literacy_Movement.pdf.

[59] Gulati S. Technology-enhanced learning in developing nations: A review. Int Rev Res Open Distance Learn. 2008; 9(1): 1–16.

[60] Booth JL, Siegler RS. Numerical magnitude representations influence arithmetic learning. Child Dev. 2008;79(4):1016–31. 10.1111/j.1467-8624.2008.01173.x 18717904

[61] Fazio LK, Bailey DH, Thompson C a, Siegler RS. Relations of different types of numerical magnitude representations to each other and to mathematics achievement. J Exp Child Psychol. 2014;123:53–72. 10.1016/j.jecp.2014.01.013 24699178

[62] Sasanguie D, De Smedt B, Defever E, Reynvoet B. Association between basic numerical abilities and mathematics achievement. Br J Dev Psychol. 2012;30(Pt 2):344–57. 10.1111/j.2044-835X.2011.02048.x 22550952

[63] Gunderson EA, Ramirez G, Beilock SL, Levine SC. The relation between spatial skill and early number knowledge: the role of the linear number line. Dev Psychol. 2012;48(5):1229–41. 10.1037/a0027433 22390659 PMC10811729

[64] Siegler RS, Booth JL. Development of numerical estimation in young children. Child Dev. 2004;75(2):428–44. 10.1111/j.1467-8624.2004.00684.x 15056197

[65] Booth JL, Siegler RS. Developmental and individual differences in pure numerical estimation. Dev Psychol. 2006;41(6):189–201. 10.1037/0012-1649.41.6.189 16420128

[66] Siegler RS, Opfer JE. The development of numerical estimation: evidence for multiple representations of numerical quantity. Psychol Sci. 2003;14(3):237–43. 12741747 10.1111/1467-9280.02438

[67] Newcombe NS, Levine SC Mix K. Thinking about quantity: The intertwined development of spatial and numerical cognition. Cognitive Sci. 2015; 6: 491–505. 10.1002/wcs.1369 26415916

[68] Lourenco SF, Bonny JW, Fernandez EP, Rao S. Nonsymbolic number and cumulative area representations contribute shared and unique variance to symbolic math competence. Proc Natl Acad Sci. 2012;109(46):18737–42. 10.1073/pnas.1207212109 23091023 PMC3503215

[69] Chen Q, Li J. Association between individual differences in non-symbolic number acuity and math performance: A meta-analysis. Acta Psychol. 2014;148:163–72. 10.1016/j.actpsy.2014.01.016 24583622

[70] Schneider M, Beeres K, Coban L, Merz S, Schmidt SS, Stricker J, et al Association of non-symbolic and symbolic numerical magnitude processing with mathematical competence: A meta-analysis. Developmental Sci. In Press 10.1111/desc.12372 26768176

