# Supplementary material for: Effects of Non-Symbolic Approximate Number Practice on Symbolic Numerical Abilities in Pakistani Children
Source: PLoS One. 2016 Oct 20;11(10):e0164436. doi: 10.1371/journal.pone.0164436 (PMC5072670; doi:10.1371/journal.pone.0164436)

S1 Appendix: Further Methodological Details

Training task gameplay

Training tasks were presented as a game in which either a good cartoon character surreptitiously added more items to a set (or made a line segment taller, or made an object lighter,) or a bad cartoon character surreptitiously stole items from the set (or made the line segment shorter, or made the object darker) while the set of items, line segment, or object was hidden behind an occluder, resulting in a final set (or line or object) that was more or less numerous (or tall or bright) than the expected outcome. The game was devised in an effort to maintain children's attention throughout the training task. Familiar male and female cartoon characters were assigned based on the sex of the child.

Training task stimuli timing details

For the approximate numerical addition training condition and the line length addition condition, trials began with the occluder presented for 500 ms. An image (dot array or line) appeared on left, was static for 1000 ms, and then moved behind occluder over the next 500 ms. After a pause of 500 ms, a second image appeared to the right of the occluder (dot array or line), was static for 1000 ms, and then moved behind the occluder over the next 500 ms. The occluder remained on the screen for 1250 ms and then disappeared to reveal an outcome (dot array or line). The outcome remained on the screen for up to 5000 ms, during which the participant was instructed to make a more or less judgment on the relevant magnitude dimension. For the brightness comparison condition, trials began with the occluder presented for 500 ms. An oval image then appeared to the left and the right of the occluder and was static for 1000 ms. Over the next 800 ms, the left side of the oval disappeared behind the occluder, leaving only the right side of the oval visible and static for another 600 ms. The right side of the oval then moved behind the occluder over the next 800 ms. After a pause 500 ms, the occluder disappeared to reveal a circle of a different brightness than the original oval. Participants were instructed to make a more or less judgment on the brightness of the circle relative to the brightness of the original oval.

Problem details for approximate numerical addition training task					
Addend 1 (dots)	Addend 2 (dots)	Actual Sum (dots)	Foil (dots)	Correct Response	Ratio
13	43	56	32	Less	0.57
26	30	56	32	Less	0.57
21	11	32	56	More	0.57
15	17	32	56	More	0.57
40	9	49	28	Less	0.57
22	27	49	28	Less	0.57
18	10	28	49	More	0.57
13	15	28	49	More	0.57
30	12	42	24	Less	0.57
19	23	42	24	Less	0.57
16	8	24	42	More	0.57
12	12	24	42	More	0.57
24	11	35	20	Less	0.57
16	19	35	20	Less	0.57
13	7	20	35	More	0.57
9	11	20	35	More	0.57
19	9	28	16	Less	0.57
13	15	28	16	Less	0.57
9	7	16	28	More	0.57
8	8	16	28	More	0.57
40	16	56	40	Less	0.71
28	28	56	40	Less	0.71
27	13	40	56	More	0.71
21	19	40	56	More	0.71
35	14	49	35	Less	0.71
23	26	49	35	Less	0.71
23	12	35	49	More	0.71
17	18	35	49	More	0.71
30	12	42	30	Less	0.71
20	22	42	30	Less	0.71
20	10	30	42	More	0.71
15	15	30	42	More	0.71
25	10	35	25	Less	0.71
16	19	35	25	Less	0.71
17	8	25	35	More	0.71
11	14	25	35	More	0.71
20	8	28	20	Less	0.71
13	15	28	20	Less	0.71
13	7	20	28	More	0.71
10	10	20	28	More	0.71

Problem details for line length addition training task					
Addend 1 (pixels)	Addend 2 (pixels)	Actual Sum (pixels)	Foil (pixels)	Correct Response	Ratio
84	84	168	96	Less	0.57
85	83	168	96	Less	0.57
41	55	96	168	More	0.57
60	36	96	168	More	0.57
68	79	147	84	Less	0.57
75	72	147	84	Less	0.57
35	49	84	147	More	0.57
60	24	84	147	More	0.57
61	65	126	72	Less	0.57
70	56	126	72	Less	0.57
32	40	72	126	More	0.57
47	25	72	126	More	0.57
33	72	105	60	Less	0.57
55	50	105	60	Less	0.57
30	30	60	105	More	0.57
35	25	60	105	More	0.57
40	44	84	48	Less	0.57
55	29	84	48	Less	0.57
20	28	48	84	More	0.57
26	22	48	84	More	0.57
84	84	168	120	Less	0.71
85	83	168	120	Less	0.71
59	61	120	168	More	0.71
71	49	120	168	More	0.71
68	79	147	105	Less	0.71
82	65	147	105	Less	0.71
40	65	105	147	More	0.71
55	50	105	147	More	0.71
57	69	126	90	Less	0.71
73	53	126	90	Less	0.71
30	60	90	126	More	0.71
50	40	90	126	More	0.71
47	58	105	75	Less	0.71
68	37	105	75	Less	0.71
35	40	75	105	More	0.71
45	30	75	105	More	0.71
42	42	84	60	Less	0.71
50	34	84	60	Less	0.71
28	32	60	84	More	0.71
30	30	60	84	More	0.71

Problem details for brightness comparison training task

Objects with eight levels of brightness were generated by holding hue and saturation constant and changing the brightness settings from 30 to 100 in Adobe Photoshop (objects with brightness values of 30, 40, 50, 60, 70, 80, 90, and 100, with saturation set as 30 across all objects, see A. in figure below). In easy brightness comparison condition, comparisons between the initial object brightness and the outcome object were 30 units apart (60 vs. 90; 60 vs. 30; 70 vs. 40; 70 vs. 100). Difficult brightness comparisons involved comparisons only 20 units apart (50 vs. 30; 50 vs. 70; 60 vs. 40; 60 vs. 80; 70 vs. 50; 70 vs. 90). See B. in figure below for schematic of comparisons.

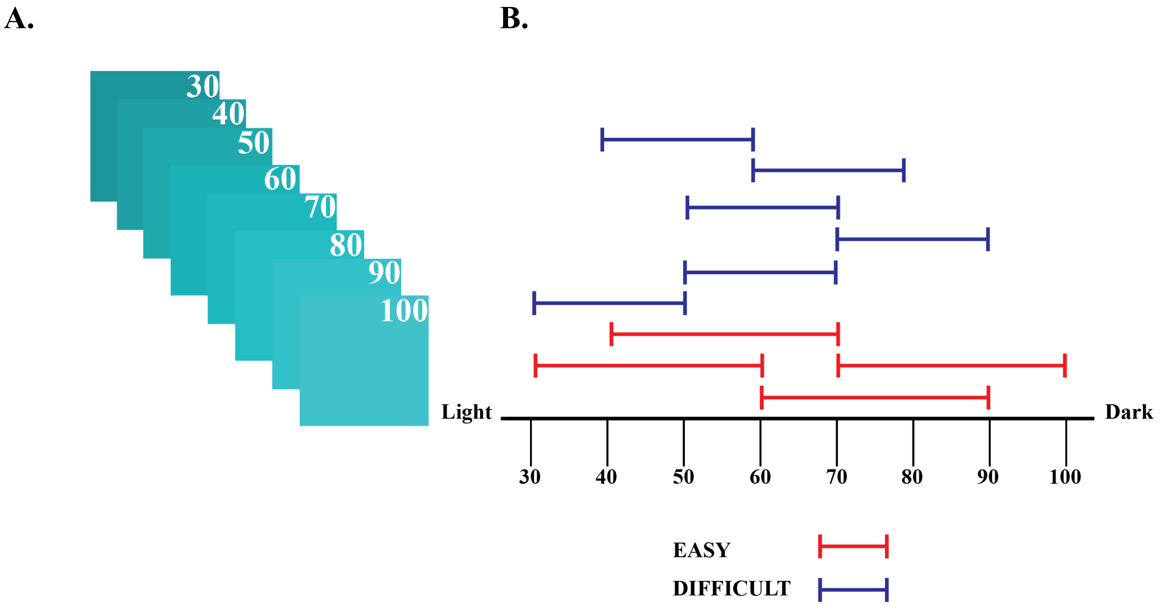

**Problem details for symbolic addition test**			
Addend 1	Addend 2	Answer	Problem Set
12	3	15	1
14	2	16	1
9	3	12	1
11	4	15	1
8	6	14	1
7	4	11	1
6	5	11	1
13	3	16	1
7	7	14	1
9	6	15	1
16	3	19	2
17	3	20	2
15	5	20	2
15	3	18	2
8	8	16	2
12	8	20	2
9	7	16	2
13	6	19	2
9	8	17	2
15	6	21	2
18	4	22	3
19	6	25	3
15	9	24	3
17	5	22	3
19	9	28	3
16	14	30	3
17	13	30	3
15	12	27	3
16	8	24	3
14	14	28	3
20	14	34	4
17	14	31	4
18	16	34	4
19	18	37	4
17	17	34	4
37	28	65	4
46	38	84	4
58	23	81	4
25	13	38	4
64	36	100	4

Sample problem for number line placement task

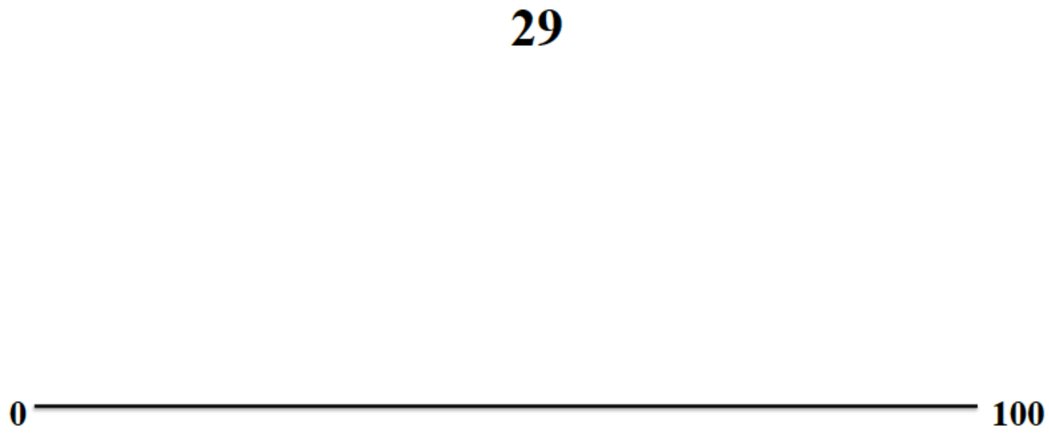

Supplement: S1 Appendix — (PDF) [file pone.0164436.s001.pdf]
